# Heart failure after pressure overload in autosomal-dominant desminopathies: Lessons from heterozygous *DES*-p.R349P knock-in mice

**DOI:** 10.1371/journal.pone.0228913

**Published:** 2020-03-03

**Authors:** Florian Stöckigt, Lars Eichhorn, Thomas Beiert, Vincent Knappe, Tobias Radecke, Martin Steinmetz, Georg Nickenig, Viktoriya Peeva, Alexei P. Kudin, Wolfram S. Kunz, Carolin Berwanger, Lisa Kamm, Dorothea Schultheis, Ursula Schlötzer-Schrehardt, Christoph S. Clemen, Rolf Schröder, Jan W. Schrickel

**Affiliations:** 1 Department of Cardiology, University Hospital Bonn, Bonn, Germany; 2 Department of Cardiology, Krankenhaus Porz, Urbacher Weg, Cologne, Germany; 3 Department of Anesthesiology, University Hospital Bonn, Bonn, Germany; 4 Department of Cardiology, University Hospital Essen, Hufelandstraße, Essen, Germany; 5 Institute of Experimental Epileptology and Cognition Research, Bonn, Germany; 6 Department of Epileptology, University Hospital of Bonn, Bonn, Germany; 7 Institute of Aerospace Medicine, German Aerospace Center (DLR), Linder Höhe, Cologne, Germany; 8 Institute of Neuropathology, University Hospital Erlangen, Friedrich-Alexander University Erlangen-Nürnberg, Schwabachanlage, Erlangen, Germany; 9 Department of Opthalmology, University Hospital Erlangen, Friedrich-Alexander University Erlangen-Nürnberg, Schwabachanlage, Erlangen, Germany; 10 Center for Biochemistry, Institute of Biochemistry I, Medical Faculty, University of Cologne, Cologne, Germany; 11 Center for Physiology and Pathophysiology, Institute of Vegetative Physiology, Medical Faculty, University of Cologne, Cologne, Germany; Heart and Diabetes Center NRW, UNiversity Hospital of the Ruhr-University Bochum, GERMANY

## Abstract

**Background:**

Mutations in the human desmin gene (*DES*) cause autosomal-dominant and -recessive cardiomyopathies, leading to heart failure, arrhythmias, and AV blocks. We analyzed the effects of vascular pressure overload in a patient-mimicking p.R349P desmin knock-in mouse model that harbors the orthologue of the frequent human *DES* missense mutation p.R350P.

**Methods and results:**

Transverse aortic constriction (TAC) was performed on heterozygous (HET) *DES*-p.R349P mice and wild-type (WT) littermates. Echocardiography demonstrated reduced left ventricular ejection fraction in HET-TAC (WT-sham: 69.5 ± 2.9%, HET-sham: 64.5 ± 4.7%, WT-TAC: 63.5 ± 4.9%, HET-TAC: 55.7 ± 5.4%; p<0.01). Cardiac output was significantly reduced in HET-TAC (WT sham: 13088 ± 2385 μl/min, HET sham: 10391 ± 1349μl/min, WT-TAC: 8097 ± 1903μl/min, HET-TAC: 5793 ± 2517μl/min; p<0.01). Incidence and duration of AV blocks as well as the probability to induce ventricular tachycardias was highest in HET-TAC. We observed reduced mtDNA copy numbers in HET-TAC (WT-sham: 12546 ± 406, HET-sham: 13526 ± 781, WT-TAC: 11155 ± 3315, HET-TAC: 8649 ± 1582; p = 0.025), but no mtDNA deletions. The activity of respiratory chain complexes I and IV showed the greatest reductions in HET-TAC.

**Conclusion:**

Pressure overload in HET mice aggravated the clinical phenotype of cardiomyopathy and resulted in mitochondrial dysfunction. Preventive avoidance of pressure overload/arterial hypertension in desminopathy patients might represent a crucial therapeutic measure.

## Introduction

The disease progression in genetically determined forms of cardiomyopathies might be aggravated by different etiological factors. In the present study we investigated the influence of pressure overload on cardiac function in a knock-in mouse model for human desminopathies, which encompass autosomal-dominant and very rare autosomal-recessive forms of cardiomyopathies and myopathies [1]. Cardiac disease manifestation comprising true cardiomyopathy as well as cardiac conduction defects and arrhythmias are the major cause of premature death in this so far not specifically treatable disease [2–6]. Cardiac involvement most often results in a dilated cardiomyopathy (DCM) phenotype. The estimated prevalence a desmin gen (*DES)* mutation in all DCM patients ranges between one and two percent [3]. However, the development of a restrictive [7], hypertrophic [8], or arrhythmogenic right ventricular cardiomyopathy [6] has also been described.

The molecular pathogenesis of desminopathies is a complex, multi-level issue depending on the structural and functional properties of the desmin protein.

The latter is a type III intermediate filament protein, which self-assembles into a three-dimensional desmin filament network, thereby interlinking neighboring myofibrils at the level of Z-discs and connecting the myofibrillar apparatus to myonuclei, mitochondria, desmosomes, and costameres [9, 10]. In the context of autosomal-dominant desminopathies, the expression of mutant desmin protein in the presence of the wild-type protein inflicts deleterious effects on the formation and maintenance of the desmin filament network, which subsequently leads to myofibrillar damage [11, 12] and pathological, desmin-positive protein aggregates in striated muscle cells [13].

The clinical phenotype in humans caused by the frequently occurring *DES* mutation p.R350P varies widely. It includes muscle weakness particularly with a limb girdle distribution and cardiac involvement consisting of conduction defects, hypertrophy, heart failure, and supraventricular as well as ventricular arrhythmias. The onset of disease is in the 3rd (male) and 4th (female) decade of life. Sudden cardiac death occurs often in the 5th decade of life in affected families [14, 15]. In a previous study using our patient-mimicking *DES*-p.R349P knock-in mouse strain (nucleotide exchange c.1045_1047delAGG>insCCC) that harbors the orthologue of the human *DES* mutation p.R350P [1], we demonstrated myofibrillar damage and desmin-positive protein aggregates as seen in human patients with this mutation. Additionally, we found that the expression of mutant desmin protein can cause widespread mitochondrial pathology comprising morphological, enzymatic and genetic alterations in skeletal muscle tissue [16]. Further exploiting this desminopathy mouse model, we now studied the impact of transverse aortic constriction (TAC)-induced pressure overload on cardiac function and mitochondrial properties.

## Methods

### Desminopathy mouse model

The generation and genotyping of the *DES*-p.R349P knock-in mouse model B6J.129Sv-*Des*^tm1.1Ccrs^ has previously been reported by our working group [1]. Heterozygous (HET) *DES*-p.R349P mice are fertile and reproduce according to Mendelian distribution. They do not show clinical signs of heart failure at younger ages. Only at the age of 24 months clinical evidence of cardiomyopathy was measurable in HET *DES*-p.R349P mice with invasive left heart catheterization [1]. We deliberately used young HET mice without spontaneous signs of cardiomyopathy and wild-type littermates (WT) for all experiments. Routine genotyping of *DES-*p.R349P knock-in mice was done by PCR using primer pair 5'-AAACCTGGAAGCAGTTTTACACAAGAGGC-3' and 5'-GCTGTAGGTTTTTAATTCTAAAGGTGGATAAGGG-3' resulting in products of 179 and 244 bp for the WT and mutant alleles, respectively. Since mice homozygous for the p.R349P missense mutation exhibited a disproportionately high mortality after TAC, they were not included in this study. All applicable international, national, and institutional guidelines for the care and use of animals were followed. The procedures were conducted in accordance with the EU directive 2010/63/EU for animal experiments. All procedures were approved by the responsible governmental animal care and use office (North Rhine-Westphalia State Agency for Nature, Environment and Consumer Protection (LANUV), Recklinghausen, Germany; reference number 84–02.04.2015.A302). All surgery was performed under inhalative anesthesia, and all efforts were made to minimize suffering.

### Transverse aortic constriction

Pressure overload to the heart was induced using TAC operation as previously described [17]. Sham (sh) operated animals served as controls. In brief, under inhalative anesthesia (1.2 vol% isoflurane in 70%N_2_O/30%O_2_), the aortic arch was dissected via lateral thoracotomy in the 2^nd^ intercostal space without entering the chest cavities. With a 6.0 suture, constriction was performed between the brachiocephalic artery and left common carotid artery using a 27 G needle as a spacer. For sham operation we followed the same protocol only without ligation of the aorta. The intercostal space and the skin were closed using 6.0 polypropylene sutures. All further experiments were carried out 14 days after TAC surgery.

### Echocardiography

Transthoracic murine echocardiography was performed utilizing a high frequency ultrasound device (Vevo 2100, VisualSonics, Fujifilm) with a high frequency transducer (MS 400, VisualSonics, Fujifilm) to determine left ventricular function in vivo. The platform and the ultrasound gel were pre-heated to 40°C. Anesthesia was induced with isoflurane at a concentration of 1 to 2 vol% with an oxygen flow of 1 l/min. After a stable sedation was confirmed, mice were gently fixed on all four limbs on the ECG electrodes for surveillance of the heart rate and a chemical hair removal was performed. The temperature was measured with a rectal thermometer. Anesthesia was adapted to maintain a heart rate of 500–600/min and a body temperature of 36.5–38.0°C during the whole examination. Recordings were performed in parasternal long and short axis views and all calculations were done from mid-ventricular derived M-mode sections using a specialized software (Vevo LAB, VisualSonics, Fujifilm).

### Left heart catheterization

For verification of pressure overload to the heart and a successful long-term TAC procedure, mice were subjected to left heart catheterization. Under inhalative anesthesia (1.2 vol% isoflurane in 70%N_2_O/30%O_2_) a pressure-volume loop catheter (Millar, SPR-839; 1.4F) was inserted in the right carotid artery and advanced to the left ventricle of the heart. Systolic and diastolic blood pressures were measured in the right common carotid artery and pressure-volume loops were recorded in the left ventricle and analyzed using LabChart 7pro (V8.1.5, AD Instruments and Millar’s PVAN pressure-volume analysis software 3.6).

### Quantification of myocardial fibrosis

Ventricular myocardial tissue was snap frozen in liquid nitrogen-cooled isopentane and cut into four-micrometer sections. Samples were stained with Sirius red (0.1% in saturated aqueous picric acid; Sigma Aldrich). Myocardial fibrosis was determined in 10 random fields of the left ventricle at 200x magnification. The proportion of extracellular Sirius red staining was calculated using digital planimetry (Adobe Photoshop, V7.0) and expressed in relation to the total myocardial slice area. In addition, standard H&E stains were performed and images were recorded using an Olympus CX41 light microscope (Olympus, Hamburg, Germany).

### Telemetric ECG recording

For detection of spontaneous arrhythmia, we used ECG telemetry devices (Modell EA-F20; DataSciences International, St. Paul, MN). The devices were implanted under inhalative anesthesia (1.2 vol% isoflurane in 70%N_2_O/30%O_2_) in a subcutaneous tissue pocket. The leads were tunneled subcutaneously and fixed to the pectoralis muscle in an Eindhoven II position. The 24-hour ECG recordings (LabChart 7pro; v7.3.3, AD Instruments) were performed 14 days after the surgical procedure in conscious and freely moving mice without the need of anesthesia. Signal averaged ECGs were calculated from 200 consecutive QRS complexes. Rate corrected QT interval (QTc) was calculated according to Mitchell et al. [18].

### Electrophysiological investigation

*In vivo* electrophysiological (EP) investigations were performed as previously described [19]. Under inhalative anesthesia (1.2 vol% isoflurane in 70% N_2_O/30% O_2_) a 2-French octapolar mouse EP catheter (Ciber Mouse, NuMed Inc., NY, USA) was transvenously inserted into the right heart chambers. We used a modified multi-programmable stimulator (Model 5328; Medtronic, MN, USA) to determine functional EP parameters. Sinus node recovery time (SNRT) was defined as the maximum return cycle length after 10 s fixed-rate atrial pacing at S1S1 cycle length 120 ms. Wenckebach periodicity (WBP) defined as the longest S1S1 cycle length with loss of 1:1 AV nodal conduction. Atrial, ventricular and AV nodal refractory periods (ARP, VRP and AVNRP) were evaluated by programmed stimulation maneuvers. Atrial burst stimulation (5 s at S1S1: 50–10 ms, 10 ms stepwise reduction; stimulus amplitudes 1.0 and 2.0 mA; resulting in a total of 10 atrial burst stimulations per animal) was used for the induction of atrial fibrillation (AF). The inducibility of ventricular tachyarrhythmias (VTs) was determined by ventricular extrastimulus pacing (S1S1: 120 ms, 100 ms, and 80 ms followed by up to 3 extra beats) and ventricular burst stimulation (1 s at S1S1: 50–10 ms, 10 ms stepwise reduction; stimulus amplitudes 1.0 and 2.0 mA, resulting in a total of 19 stimulations per animal). VT was defined as ≥ 4 consecutive ventricular ectopic beats. The probability for the induction of AF and VT episodes was determined by dividing the total number of arrhythmia episodes in each group by the total number of test maneuvers performed in this group.

### mtDNA long-range PCR (LR-PCR)

Total DNA from left ventricular muscle was isolated through column purification with QIAamp DNA Mini Kit (QIAGEN N.V., Venlo, Netherlands) as described by the manufacturer. Each sample was eluted in 200 μl elution buffer provided with the kit and stored without freezing at 4°C. LR-PCR was used in order to detect mtDNA deletions. For that purpose, almost the entire mtDNA was amplified between primers musMT2482F (5’-GTTCAACGATTAAAGTCCTACGTG-3’) and musMT1005R (5’-CCAGTATGCTTACCTTGTTACGAC-3’) (first number, 5’-end of the primer; F-forward or R-reverse) by the use of TaKaRa LA Taq Hot Start polymerase (Clontech). The LR-PCR was performed under the following conditions: 95°C for 2.5 min, 30 cycles of 92°C for 20 s and 66.8°C for 5:30 min, and 72°C for 10 min. The PCR products were loaded on a 1% agarose gel with Quick-Load 1 kb Extend DNA Ladder (New England Biolabs, NEB).

### mtDNA copy number

Quantitative real-time PCR (qPCR) was used for the mtDNA copy number determination, using total DNA, prepared as described above. The qPCR was performed with 2×SYBR Green qPCR Master Mix (Bimake, Munich, Germany) and three different DNA concentrations, with final DNA amounts of: 5 ng, 10 ng and 20 ng. Each sample was used in triplicates for each dilution. The primers amplifying the murine mitochondrial fragment were musMT553F (5’-GCCAGAGAACTACTAGCCATAGC-3’) and musMT668R (5’-AGCAAGAGATGGTGAGGTAGAGC-3’) (first number, 5’-end of the primer; F-forward or R-reverse). A single copy gene encoding the inward rectifier potassium channel 13 (*Kcnj13*) was used as a nuclear reference gene. The primer pair for *Kcnj13* was mus4987F (5’-GGATGAGAGAGAGAAGCACAAGTGG-3’) and mus5140R (5’-CTGTATGACCAACCTTGGACATGAT-3’) (first number, 5’-end of the primer; F-forward or R-reverse). All primer pairs were PCR optimized and checked by PAGE. The qPCRs were performed under the following conditions: 95°C for 7:00 min, 45 cycles of 95°C for 15 s and 62.6°C (nuc gene) or 64.6°C (mito gene) for 1 min, 95°C for 1 min and 55°C for 1 min.

SigmaPlot (2001 for Windows version 7.0, Systat Software GmbH) and Chapman sigmoidal non-linear regression curve fitting was used for the analysis of the obtained qPCR fluorescence data [20]. The parameters *y*_*0*_, *a*, *b* and *c* determining the shape of the sigmoidal regression curve *y* = *y*_*0*_ + *a*(1 –*e*^*-bx*^*)*^*c*^ were obtained from the fitting program. The *C*_*t*_ values for the mtDNA and the nuclear reference gene were calculated from the inflection point of the sigmoidal curve using the equation *C*_*t*_ = ln(*c*)/*b*. These values were used for the calculation of the mtDNA copy number according to Zsurka et al.[21]. By subtracting the *C*_*t*_ values of the mtDNA fragment (*C*_*t*_mito) from the *C*_*t*_ values of the reference gene (*C*_*t*_nuc), the cycle number difference (Δ*C*_*t*_) was calculated (Δ*C*_*t*_ = *C*_*t*_nuc − *C*_*t*_mito). The copy number (CN) of the mtDNA relative to the diploid single nuclear gene was calculated as CN = 2 × 2^ΔCt^. Additionally, the PCR amplification efficiency for each primer pair was determined (PCR efficiency = (10^−1/slope^ − 1) × 100) [22]; 103% for musMT553F/musMT668R, and 95% for mus4987F/mus5140R (Kcnj13)[16].

### Respiratory complex I and IV activities

Ventricular cardiac tissue specimens snap-frozen in liquid nitrogen were homogenized three times for 15 s at 24,000 rpm (Ultra-Turrax homogenizer (IKA, Staufen, Germany)) in 0.1 M phosphate buffer with pH 7.4 (1 ml buffer per 25 mg tissue) and centrifuged at 16,000×*g* for 15 min at 4°C as previously described [23]. The supernatants, containing all cytosolic (99–100% of lactic dehydrogenase; tested in six independent control samples) and all mitochondrial matrix enzymes (90–95% of citrate synthase; tested in six independent control samples) were kept snap-frozen in liquid nitrogen and later used for determination of citrate synthase, see below. The pellets were re-suspended in half of the volume of the initially added phosphate buffer and immediately used for the measurement of the inner membrane associated enzyme activities (99–100% of cytochrome *c* oxidase and NADH:CoQ1 reductase). A dual wavelength spectrophotometer (Aminco DW 2000, SLM Instruments, Rochester, NY, USA) at 340/380 nm (ε_red-ox_ = 5.5 mM^-1^ cm^-1^) was used for the measurement of the rotenone-sensitive NADH:CoQ 1 oxidoreductase (complex I) activity; the measurement was performed at 30°C. The assay was initiated by the sample addition to a reaction mix, containing 50 mM KCl, 1 mM EDTA, 10 mM Tris-HCl pH 7.4, 1 mM KCN, 100 μM CoQ_1_, and 150 μM NADH, followed by monitoring of the velocity of NADH oxidation. 20 μM rotenone was added to the assay mixture after 2 min in order to determine the rotenone-insensitive NADH oxidation rate. The complex I activities were determined from the differences of the total NADH oxidation rate and the rotenone-insensitive NADH oxidation rate. The cytochrome *c* oxidase (complex IV) activities were measured by monitoring the oxidation of ferrocytochrome c in its β-band at the wavelength pair 510/535 nm (ε_red-ox_ = 5.9 mM^-1^ cm^-1^). The measurement was performed at 30°C in 0.1 M potassium phosphate buffer (pH 7.4) containing 0.02% laurylmaltoside (Sigma-Aldrich, St. Louis, Missouri, USA). In order to obtain reduced cytochrome *c*, oxidized bovine heart cytochrome *c* (purity 99%, Sigma-Aldrich, St. Louis, Missouri, USA) was reduced with ascorbate, desalted on a Sephadex-G25 column, and stored in liquid nitrogen until use.

### Citrate synthase activity

Citrate synthase activity was determined by the reduction of 5’,5’-Dithiobis-(2-nitrobenzoic acid) (DTNB) by CoA-SH liberated by the citrate synthase reaction in the presence of oxaloacetate and acetyl-CoA as described previously [24, 25]. The incubation mix contained 720 μl H_2_O, 100 μl 0.1 mM DNTB (Sigma-Aldrich, St. Louis, Missouri, USA; in 1 M Tris-HCl pH 8.1), 25 μl 10% Triton X-100, 50 μl 10 mM oxalacetate (Sigma-Aldrich, St. Louis, Missouri, USA; in 0.1 M triethanolamine-HCl pH 8.0 with 5 mM EDTA), and 25 μl 12.2 mM acetyl-CoA (Sigma-Aldrich, St. Louis, Missouri, USA). The samples were diluted 1:2 to 1:6 in 0.1 M Tris-HCl pH 7.0, and 80 μl samples were added to 920 μl of incubation mix in a plastic cuvette. The absorbance change at 412 nm was monitored in a dual wavelength spectrophotometer (Aminco DW 2000, SLM Instruments, Rochester, NY, USA).

### Ultrastructural analysis

Transmission electron microscopy was performed according to [1]. Left ventricular cardiac muscle specimens were fixed in 2.5% glutaraldehyde in 0.1 M phosphate buffer, pH 7.2, postfixed in 2% buffered osmium tetroxide, dehydrated in graded alcohol concentrations, and embedded in epoxy resin according to standard protocols. Ultra-thin sections were stained with uranyl acetate and lead citrate, and examined with a LEO 906E transmission electron microscope (Carl Zeiss GmbH, Oberkochen, Germany).

### Statistical analysis

Data analysis and calculations were performed using GraphPad Prism (version 5.0b) and values in the manuscript were expressed as mean ± standard deviation. We performed the Kolmogorov-Smirnov test for Gaussian distribution for all values. Whenever the values passed the normality test we used One-way ANOVA, otherwise the nonparametric Kruskal-Wallis test for statistical analysis. Differences between individual groups were assessed using Tukey’s or Dunn’s multiple comparison test, respectively. Discrete variables were analyzed by two-sided Fisher’s exact test. A *p*-value < 0.05 was regarded as statistically significant.

## Results

### Morphological and functional alterations after pressure overload

**Fig 1:**
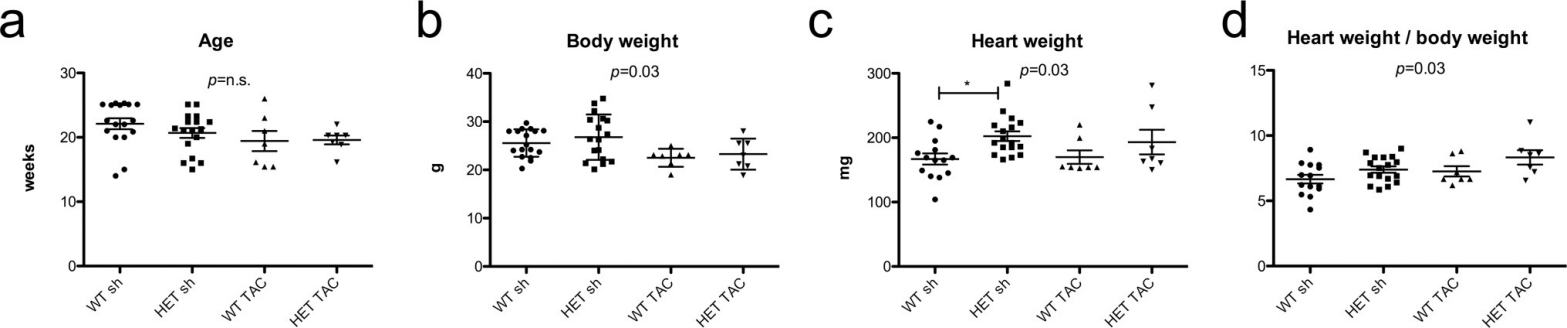
Physiological parameters. a) All experiments were performed in adult, approximately 20 weeks old mice. b) TAC operation resulted in a reduced body weight. c) TAC operation did not alter the heart weights in both genotypes. d) The heart weight / body weight ratio was highest in HET-TAC mice indicating cardiac hypertrophy. WT sh: 8 male, 7 female, mean age 22.1 weeks; HET sh: 8 male, 7 female, mean age 20.7 weeks; WT TAC: 4 male, 3 female, mean age 19.4 weeks; HET TAC: 4 male, 3 female, mean age 19.6 weeks; * = p<0.05 with Tukey’s Multiple Comparison Test.

All experiments and readouts were performed in adult mice (WT sh: 22.1 ± 3.5 weeks; HET sh: 20.7 ± 3.1 weeks; WT TAC: 19.4 ± 4.1 weeks; HET TAC: 19.6 ± 1.8 weeks; *p* = n.s.) equally divided between both sexes two weeks after TAC or sham operation, respectively (Fig 1). Survival two weeks after TAC operation was 85% in WT animals and 82% in HET animals. Body weight in mice after TAC operation was lower than in mice that underwent a sham procedure (WT TAC: 22.5 ± 1.9 g; HET TAC: 23.3 ± 3.2 g; WT sh: 25.6 ± 2.8 g; HET sh: 26.8 ± 4.7 g; p = 0.034). HET sh mice showed higher heart weights compared to WT sh mice (HET TAC 193.2 ± 50.5 mg, WT TAC 169.9 ± 27.9 mg; HET sh 202.5 ± 30.8 mg, WT sh 167.0 ± 31.9 mg; p = 0.029). TAC operation per se did not result in heart weight changes in both genotypes. As a result, the group of HET TAC animals had the highest heart weight / body weight ratio among all groups (HET TAC: 8.3 ± 1.4, WT TAC: 7.2 ± 1.0, HET sh: 7.5 ± 0.9, WT sh: 6.7 ± 1.2; p = 0.02), thus indicating the presence of cardiac hypertrophy. The tibia length of all mice did not differ between the groups. Consistent with the previous results, the calculated heart weight / tibia length ratio also showed the highest ratio in HET TAC animals (HET TAC: 11.2 ± 0.9, WT TAC: 10.1 ± 0.8, HET sh: 9.7 ± 0.8, WT sh: 9.4 ± 1.1; p = 0.03) (S1 Fig).

**Fig 2:**
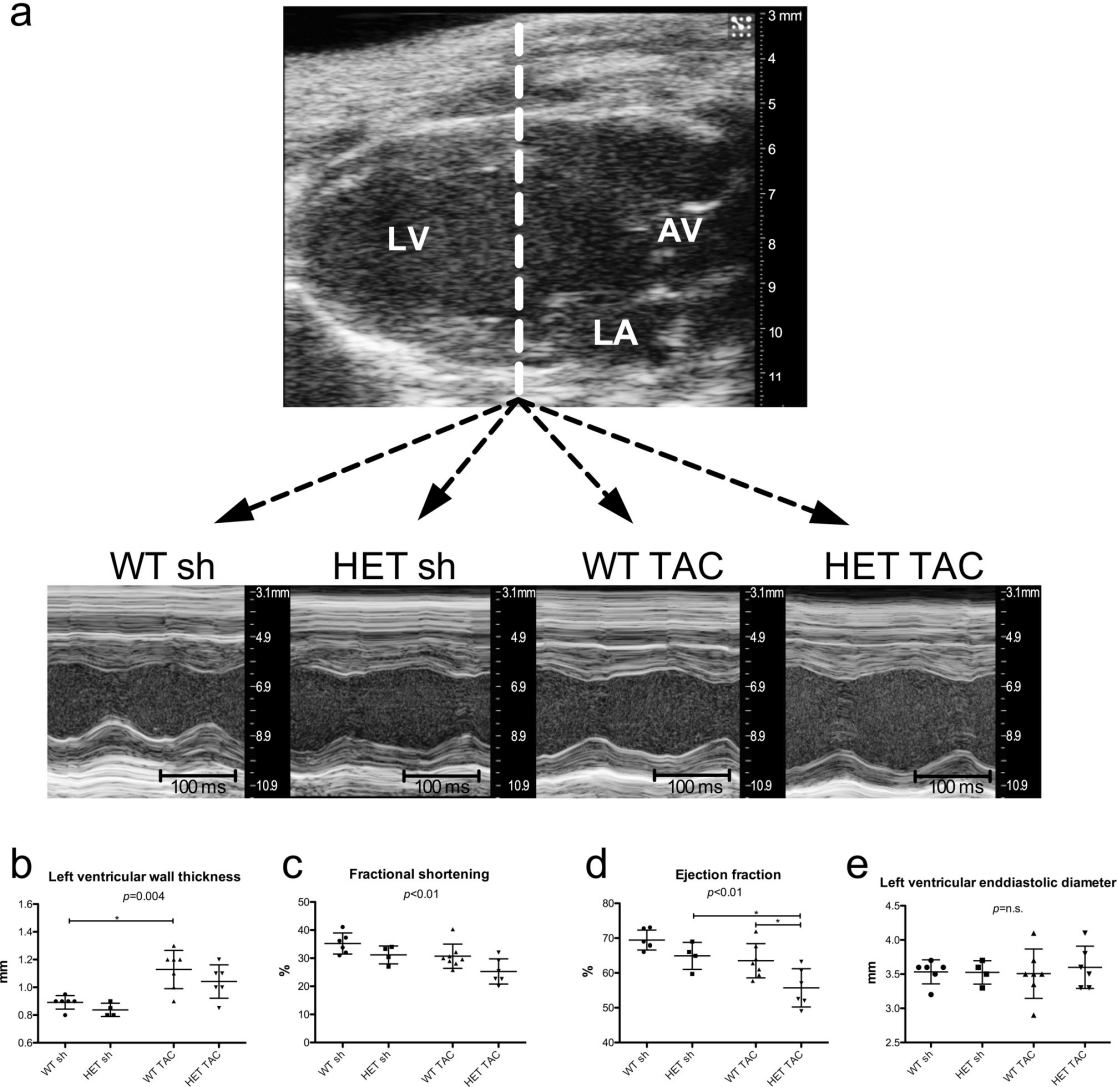
Echocardiography. a) Echocardiographic parameters were analyzed in M-mode sections of the heat. b) The left ventricular end-diastolic wall thickness was increased in both TAC groups, though only WT TAC mice reached statistical significance in Dunn’s Multiple Comparison Test. c) Fractional shortening was lowest in HET TAC. d) A significant reduction of ejection fraction after TAC was noted only in HET, but not in WT mice. e) Left ventricular end-diastolic diameter did not differ between the groups. LV = left ventricle, LA = left atrium, AV = aortic valve. WT sh: 3 male, 3 female, mean age 21.1 weeks; HET sh: 2 male, 2 female, mean age 20.5 weeks; WT TAC: 4 male, 3 female, mean age 19.4 weeks; HET TAC: 3 male, 3 female, mean age 20.2 weeks; * = p<0.05 with Dunn’s Multiple Comparison Test in b) and Tukey’s Multiple Comparison Test in b), c) + e).

Using transthoracic echocardiography, we could demonstrate increased left ventricular end-diastolic wall thickness in both TAC groups, which shows, that mutant mice are capable of responding to pressure overload with hypertrophy (Fig 2). HET TAC mice exhibited the lowest values for left ventricular fractional shortening (HET TAC: 25.3 ± 4.5%, WT TAC: 30.8 ± 4.6%, HET sh 31.2 ± 3.2%, WT sh: 35.2 ± 3.7%; p < 0.01) and reduced left ventricular ejection fraction (HET TAC: 55.7 ± 5.4%, WT TAC: 63.5 ± 4.9%, HET sh: 64.9 ± 3.9%, WT sh: 69.5 ± 2.9%; p < 0.01). This corresponded to a decrease in ejection fraction after TAC by 13.7% in HET mice and by only 8.6% in WT mice. The left ventricular end-diastolic diameters did not differ between the groups (WT sh: 3.5 ± 0.2 mm, HET sh: 3.5 ± 0.2 mm, WT TAC: 3.5 ± 0.4 mm, HET TAC: 3.6 ± 0.3 mm; *p* = n.s.).

**Fig 3:**
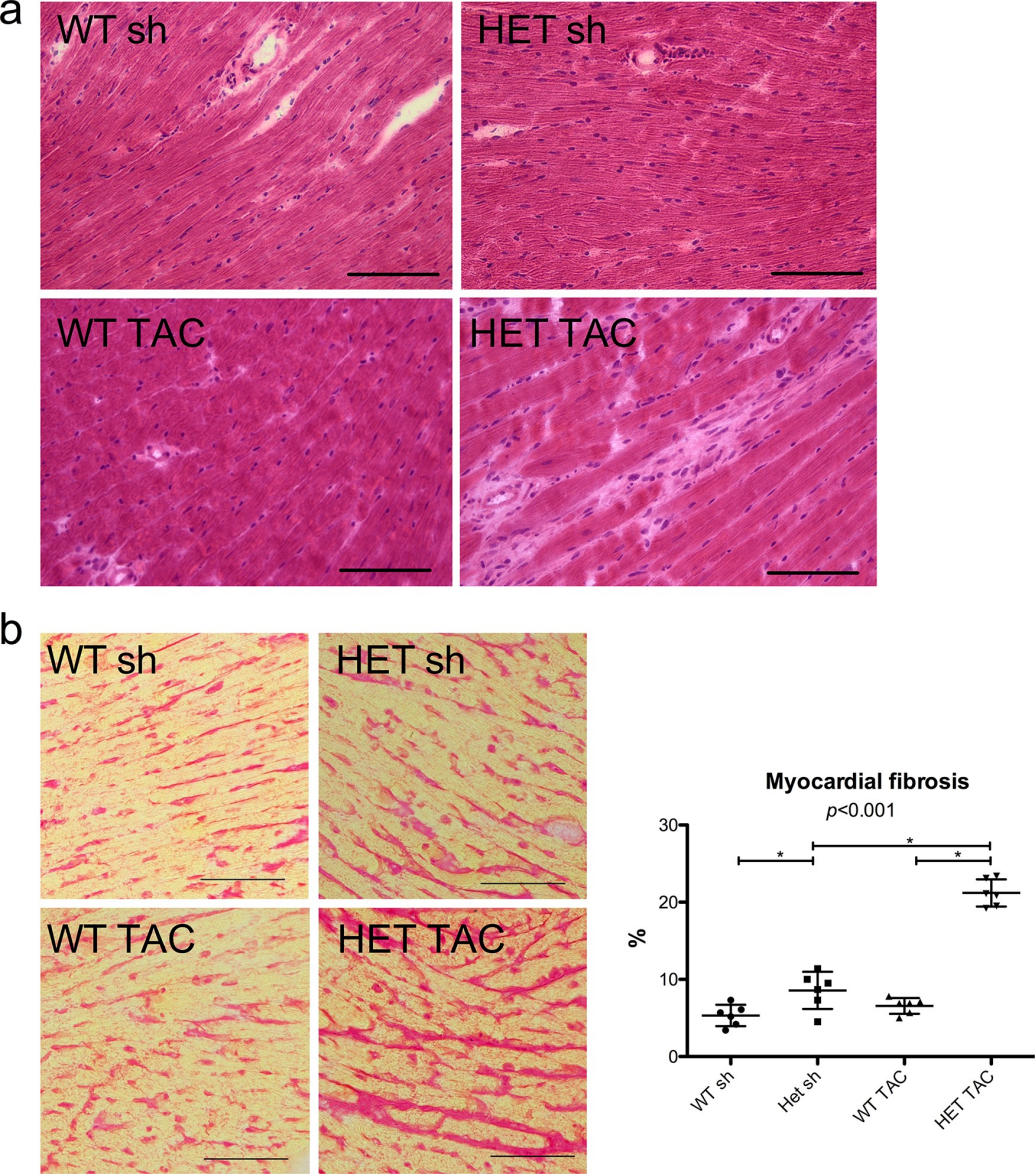
Myocardial fibrosis. a) Representative H&E stainings of all groups show the accumulation of interstitial fibrosis in HET TAC animals. b) The percentage of extracellular fibrosis as determined by Sirius red staining was significantly raised in HET TAC as compared to HET sh and WT TAC. Bar = 100μm. WT sh: 3 male, 3 female, mean age 20.0 weeks; HET sh: 3 male, 3 female, mean age 21.1 weeks; WT TAC: 3 male, 3 female, mean age 19.9 weeks; HET TAC: 3 male, 3 female, mean age 20.7 weeks; * = p<0.05 with Tukey’s Multiple Comparison Test.

Histopathological analysis of heart sections revealed a marked increase in intercellular fibrosis in HET TAC mice as compared to HET sh and WT TAC animals after 14 days (WT sh 5.3% ± 1.4, HET sh 8.6 ± 2.4%, WT TAC 6.6 ± 1.0%, HET TAC 21.2 ± 1.8%; p < 0.001) (Fig 3).

### Mutant desmin protein is associated with hemodynamic deterioration after TAC

**Fig 4:**
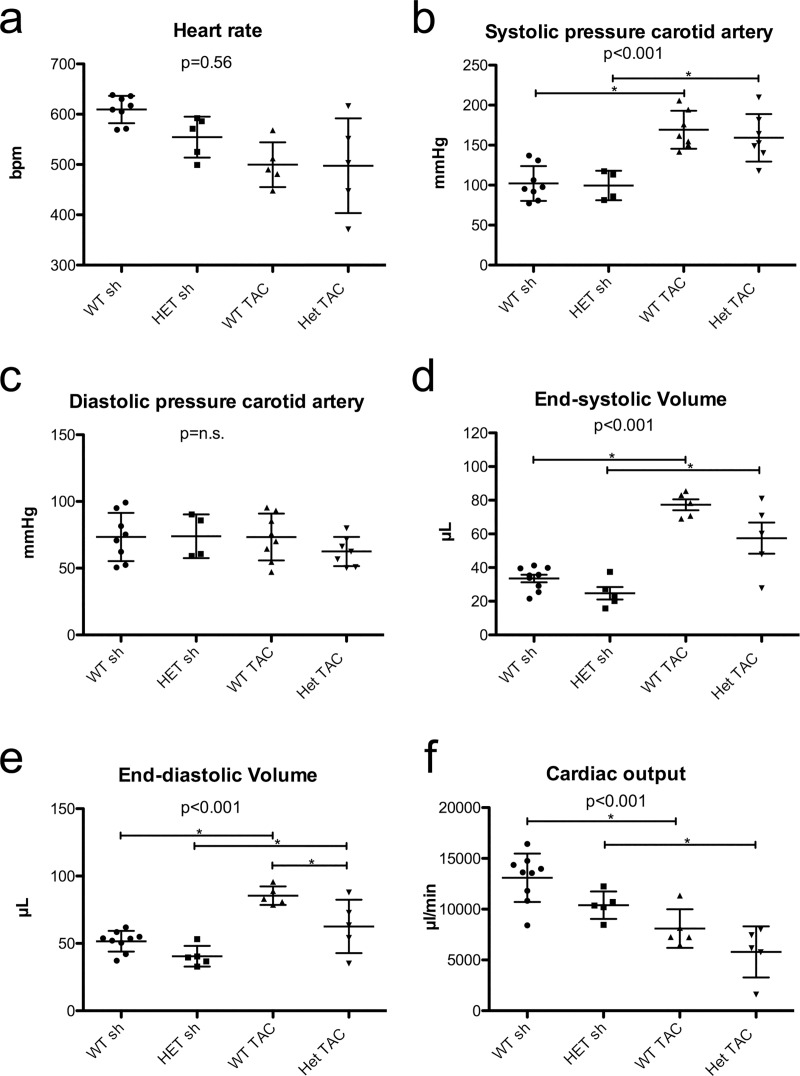
Hemodynamics. a) Left heart catheterizations with a Millar catheter were performed at equal heart rates in all groups. b+c) Systolic and diastolic carotid pressures were significantly increased after TAC in both groups indicating an effective TAC procedure. d+e) Likewise, end-systolic and end-diastolic volumes were elevated after TAC in both groups. f) Cardiac output decreased in HET TAC and WT TAC as compared to the sham groups. WT sh: 5 male, 4 female, mean age 20.4 weeks; HET sh: 3 male, 2 female, mean age 21.0 weeks; WT TAC: 4 male, 4 female, mean age 19.9 weeks; HET TAC: 3 male, 4 female, mean age 20.5 weeks n = 9 for WT sh, n = 5 for WT TAC, HET sh, and HET TAC; * = p<0.05 with Tukey’s Multiple Comparison Test.

To investigate true hemodynamic responses after TAC, we subjected all groups to *in vivo* left heart catheterization using a pressure volume catheter (Fig 4). Under equal baseline conditions (mean heart rate in all groups 549.3 ± 70.7 bpm) TAC procedure led to a significant increase in systolic carotid pressure (increase from 102.1 ± 21.8 mmHg to 169.2 ± 23.8 mmHg in WT and from 99.5 ± 18.5 mmHg to 159.2 ± 29.8 mmHg in HET; p < 0.01). Diastolic carotid pressure did not change after TAC. Furthermore, TAC procedure led to significant increases in left ventricular volumes in both groups *per se*, although the difference was less pronounced in HET mice. The left ventricular end-systolic volume increased from 33.5 ± 6.8 μl to 77.3 ± 7.3 in WT mice and from 24.8 ± 8.2 μl to 57.5 ± 20.7μl in HET mice (p < 0.01). Similarly, the left ventricular end-diastolic volumes increased from 51.6 ± 7.7 μl to 85.4 ± 6.9 μl in WT mice and from 40.5 ± 7.6 μl to 62.6 ± 19.8 μl in HET mice (p < 0.01).

Cardiac output, taking into account the stroke volume and the individual heart rates, decreased by 38.4% after TAC in WT mice (from 13088 ± 2385 μl/min to 8097 ± 1903 μl/min; p <0.05; Tukey’s multiple comparison test) and by 44.3% in HET mice (from 10391 ± 1349 μl/min to 5793 ± 2517 μl/min; p<0.05; Tukey’s multiple comparison test) which shows that presence of mutant desmin protein leads to aggravated deterioration of cardiac function after cardiac pressure overload.

### Proarrhythmic effects after TAC

**Fig 5:**
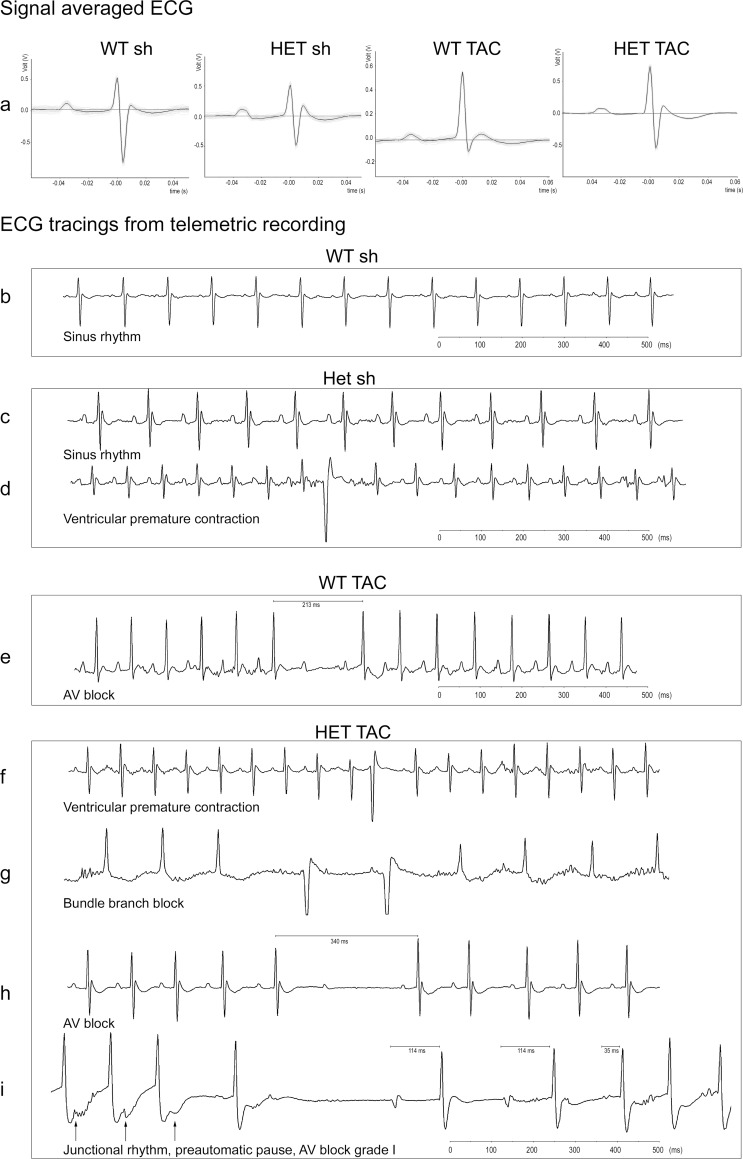
Telemetric ECG recordings. a) Signal averaged ECGs (200 QRS complexes) showed no differences in PQ, QRS, or QTc intervals between WT and HET animals. b) WT sh always showed sinus rhythm without relevant arrhythmias. c) HET sh also exhibited sinus rhythm with intermittent ventricular premature contractions (VPC) (d). e) TAC procedure in WT resulted in intermittent short-lasting AV blocks (max. 1 missing QRS complex). TAC in HET animals resulted in VPCs (f), intermittent bundle branch blocks (g), longer AV blocks (h), and accelerated junctional rhythms (arrows pointing towards retrograde P waves) and AV block grade I (i). WT sh: 1 male, 2 female, mean age 22.3 weeks; HET sh: 2 male, 1 female, mean age 19.8 weeks; WT TAC: 2 male, 1 female, mean age 20.0 weeks; HET TAC: 1 male, 2 female, mean age 19.5 weeks.

Telemetric ECG recordings in conscious animals during periods of rest demonstrated higher spontaneous heart rates after TAC operation in WT (WT sh 439.3 ± 24.1 bpm vs. WT TAC 507.5 ± 11.8 bpm; p = 0.04) compared with HET animals (HET sh 465.0 ± 27.4 bpm vs. HET TAC 477.7 ± 5.0 bpm, p = n.s.) (Table 1). We did not find differences in cardiac conduction times including PR interval, P duration, QRS interval, and QT interval (Fig 5A).

**Table 1:** Telemetric ECG recordings.

		WT sh	HET sh	WT TAC	HET TAC	*p*
24h recording						
	Heart rate (BPM)	591.3 ± 26.2	592.0 ± 27.3	648.5 ± 6.3	598.0 ± 32.6	n.s.
	PR interval (ms)	35.7 ± 0.9	37.6 ± 2.5	34.7 ± 2.6	34.8 ± 1.8	n.s.
	P duration (ms)	11.7 ± 0.2	11.8 ± 0.5	11.3 ± 1.0	13.2 ± 1.4	n.s.
	QRS interval (ms)	10.7 ± 0.5	11.0 ± 0.8	10.9 ± 0.8	13.6 ± 1.8	n.s.
	QT interval (ms)	42.3 ± 3.4	44.9 ± 2.9	44.3 ± 3.9	53.5 ± 6.4	n.s.
	QTc (ms)	40.8 ± 3.1	44.5 ± 1.8	45.7 ± 4.7	52.3 ± 5.5	n.s.
Night activity						
	Heart rate (BPM)	737.6 ± 8.2	733.7 ± 18.2	712.7 ± 10.2	679.5 ± 44.5	n.s.
	PR interval (ms)	34.7 ± 0.9	33.6 ± 2.4	32.3 ± 2.4	31.5 ± 0.5	n.s.
	P duration (ms)	10.6 ± 0.5	12.0 ± 0.8	11.1 ± 0.8	12.7 ± 0.5	n.s.
	QRS interval (ms)	11.0 ± 0.8	10.3 ± 0.5	11.7 ± 0.5	13.7 ± 2.1	n.s.
	QT interval (ms)	37.1 ± 1.4	42.0 ± 3.3	42.1 ± 2.8	53.0 ± 8.5	n.s.
	QTc (ms)	41.1 ± 2.2	46.3 ± 2.9	46.3 ± 3.8	56.3 ± 7.3	n.s.
Day rest						
	Heart rate (BPM)	439.3 ± 24.1	465.0 ± 27.4	507.5 ± 11.8*	477.7 ± 5.0	0.04
	PR interval (ms)	36.5 ± 2.1	37.3 ± 0.9	37.3 ± 0.9	36.0 ± 1.0	n.s.
	P duration (ms)	12.1 ± 0.8	11.7 ± 0.5	12.6 ± 1.2	12.7 ± 1.2	n.s.
	QRS interval (ms)	11.3 ± 1.2	10.3 ± 0.5	11.0 ± 0.1	14.7 ± 2.5	n.s.
	QT interval (ms)	47.7 ± 5.4	48.1 ± 3.7	46.3 ± 4.9	54.0 ± 4.3	n.s.
	QTc (ms)	40.6 ± 4.0	42.7 ± 1.7	42.7 ± 5.0	48.3 ± 3.9	n.s.

'Night activity' was separately analyzed during periods of nighttime activity. 'Day rest' was recorded during periods of rest at daytime. QTc: rate corrected QT time according to Miller et al.

*: p < 0.05 WT sh vs. WT TAC (Tukey’s multiple comparison test)

**Fig 6:**
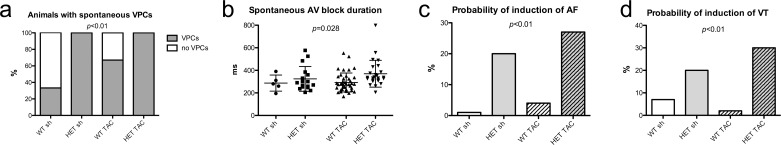
Spontaneous and inducible arrhythmias. a) During telemetric recordings only 33% of WT sh mice showed ventricular premature contractions (VPCs). After TAC this ratio increased to 67%. Spontaneous VPCs were present in all HET mice, both in the sham and TAC group. p calculated with Fisher´s exact test. b) Spontaneous AV blocks were present in all mice. However, HET TAC mice exhibited significantly longer pauses compared to WT TAC. * = p<0.05 with Tukey’s Multiple Comparison Test. c) The probability to induce atrial fibrillation during an electrophysiological study was highest in HET TAC. p calculated with Fisher´s exact test. d) Similarly, the probability to induce ventricular tachycardias was most pronounced in HET TAC. p calculated with Fisher´s exact test. for a) and b): WT sh: 1 male, 2 female, mean age 22.3 weeks; HET sh: 2 male, 1 female, mean age 19.8 weeks; WT TAC: 2 male, 1 female, mean age 20.0 weeks; HET TAC: 1 male, 2 female, mean age 19.5 weeks. for c) and d): WT sh: 4 male, 3 female, mean age 22.0 weeks; HET sh: 3 male, 4 female, mean age 21.6 weeks; WT TAC: 4 male, 3 female, mean age 19.4 weeks; HET TAC: 4 male, 3 female, mean age 19.6 weeks.

Exemplary ECG recordings from all groups are demonstrated in Fig 5B–5H. All WT mice exhibited sinus rhythm during the whole recording time. WT sh animals only rarely exhibited ventricular premature contractions (VPCs), and occasional AV blocks were restricted to singular absent QRS complexes. The incidence of mice with spontaneous VPCs was significantly higher in HET mice irrespective of sham or TAC procedure (WT sh 33%, HET sh 100%, WT TAC 67%, HET TAC 100%; p < 0.001) (Fig 6A). The mean duration of spontaneous AV blocks was longest in HET TAC mice (WT sh 287.2 ± 71.5 ms, HET sh 324.0 ± 109.3 ms, WT TAC 292.2 ± 84.0 ms, and HET TAC 370.0 ± 118.9 ms; p = 0.023) (Fig 6B). Moreover, only HET TAC mice exhibited episodes of accelerated junctional rhythm differing from sinus rhythm (Fig 5I).

In an *in vivo* electrophysiological investigation, we did not find differences in sinus- or AV-node function, and atrial or ventricular refractory periods. The probability of induction of atrial fibrillation was already increased in HET sh animals as previously described [1] and highest in HET TAC mice (WT sh 1%, HET sh 20%, WT TAC 4%, HET TAC 27%; p < 0.001; Fisher’s exact test) (Fig 6C). In WT mice, the probability of induction of VT was low after sham procedure (7%) and after TAC (2%), whereas in HET, the probability increased from 20% in sham to 30% in TAC animals (p < 0.001; Fischer’s exact test) (Fig 6D).

### Reduced mtDNA copy numbers and mitochondrial enzyme activities in HET TAC mice

**Fig 7:**
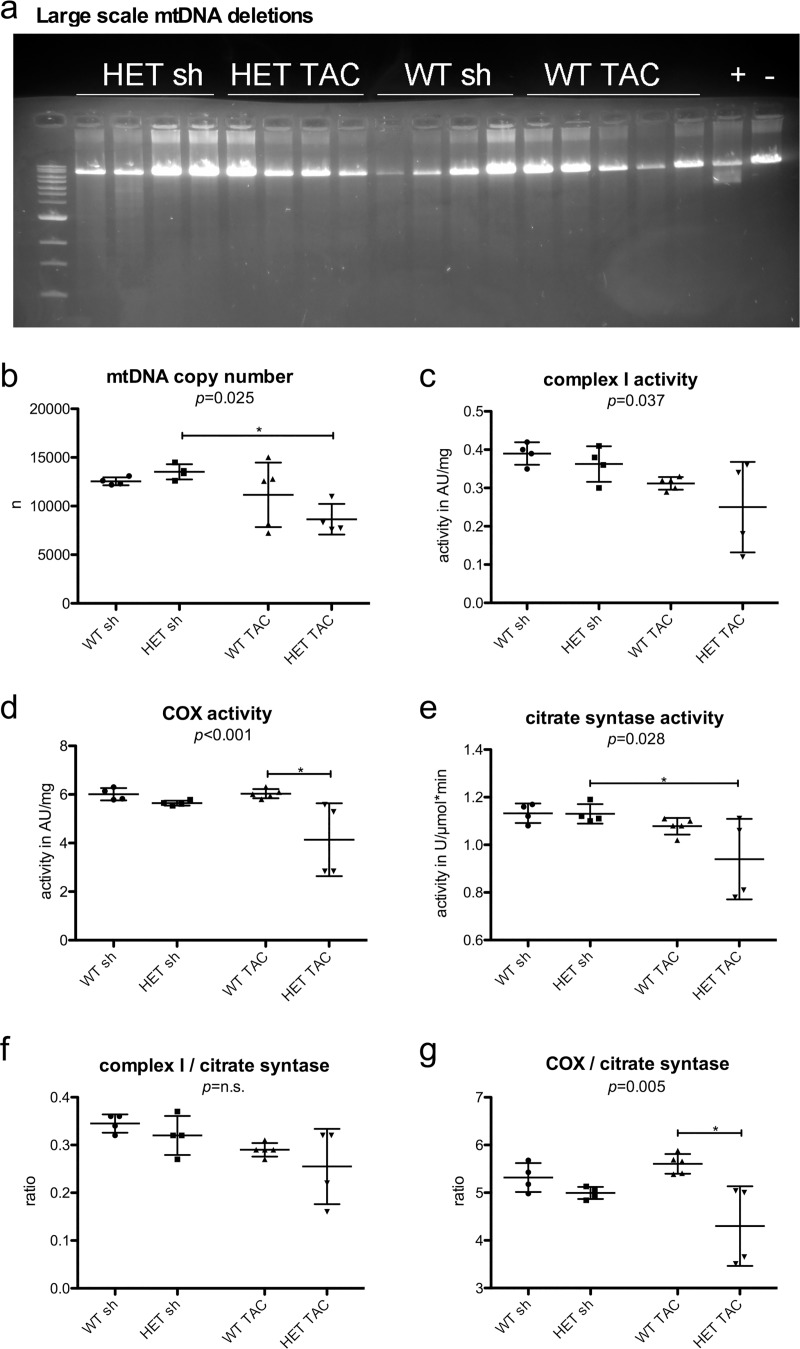
Mitochondrial DNA analyses and mitochondrial enzyme activities. a) Using long-range PCR from total DNA extracted from samples of left ventricular myocardium, we could exclude mtDNA deletions in all investigated groups. Soleus muscle from a homozygous *DES*-p.R349P mouse served as positive control for the presence of mtDNA deletions [16]. “+”: positive control; “-“: negative control. b) mtDNA copy numbers were analyzed by quantitative real time PCR. We found a significant reduction of mtDNA copy numbers in HET TAC compared to HET sh. c) Complex I activity, that was determined using a photometric assay, was significantly reduced in HET TAC animals. d) The activity of complex IV (COX) was significantly reduced only in HET TAC. e) Analogously, the determination of citrate synthase activity was reduced in HET TAC. f) The normalization of complex I activity to the level of citrate synthase activity resulted in equal values with only a slight trend towards lower values in HET TAC. g) The normalization of COX activity to the level of citrate synthase activity resulted in the lowest ratio in HET TAC. WT sh: 2 male, 2 female, mean age 20.3 weeks; HET sh: 2 male, 2 female, mean age 20.8 weeks; WT TAC: 2 male, 3 female, mean age 19.2 weeks; HET TAC: 2 male, 2 female, mean age 20.5 weeks. p calculated by 1way ANOVA, * = p<0.05 with Tukey’s Multiple Comparison Test.

Using long-range PCR we were able to exclude the presence of mtDNA deletions in all groups (Fig 7A). We determined mtDNA copy numbers by quantitative real time PCR and found significant reductions limited to the HET TAC group (WT sh 12,546 ± 606, HET sh 13,526 ± 781, WT TAC 11,155 ± 3,315, HET TAC 8,649 ± 1,582; p = 0.025) (Fig 7B). In a next step, we performed biochemical analyses that demonstrated a significant decrease in the activity of respiratory chain complex I also restricted to the group of HET TAC animals (WT sh 0.39 ± 0.03 AU/mg, HET sh 0.36 ± 0.05 AU/mg, WT TAC 0.31 ± 0.02 AU/mg, HET TAC 0.25 ± 0.12 AU/mg; p = 0.037) (Fig 7C). Similarly, the activity of COX was only reduced in HET TAC animals, as compared to WT TAC (WT sh 6.01 ± 0.25 AU/mg, HET sh 5.65 ± 0.1 AU/mg, WT TAC 6.03 ± 0.19 AU/mg, HET TAC 4.14 ± 1.5 AU/mg; p < 0.001) (Fig 7D). In addition, the citrate synthase activity also showed a significant reduction in HET TAC mice (WT sh 1.13 ± 0.04 U/μmol*min, HET sh 1.13 ± 0.4 U/μmol*min, WT TAC 1.08 ± 0.03 U/μmol*min, HET TAC 0.94 ± 0.17 U/μmol*min; p = 0.028) (Fig 7E). In keeping with the reduced mtDNA copy number, the latter findings indicate a reduction in the total amount of mitochondria after TAC operation in HET mice. The additional normalization of the complex I activity to the citrate synthase activity resulted in equal values across all four groups (Fig 7F). However, the normalization of COX to the citrate synthase activity (Fig 7G) revealed the lowest ratio in HET TAC mice (WT sh 5.3 ± 0.3, HET sh 5.0 ± 0.1, WT TAC 5.6 ± 0.2, HET TAC 4.3 ± 0.8; p = 0.005), implying that specific mitochondrial respiratory chain defects accompany the decreased mitochondrial amount in the HET TAC animals.

**Fig 8:**
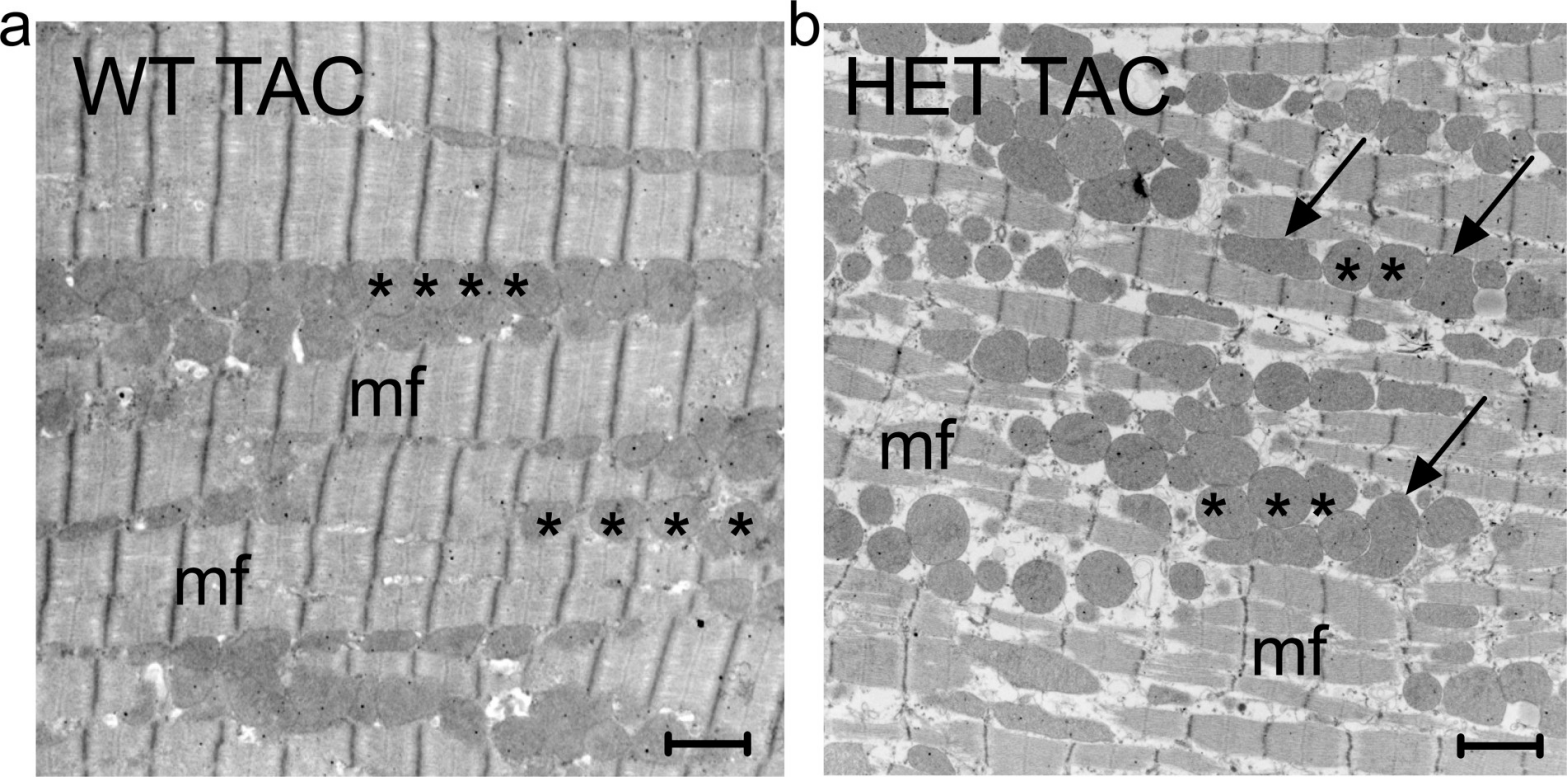
Electron microscopy. Mitochondria are arranged in a string-of-pearls-like manner alongside the myofibrils in WT TAC mice (a). In contrast, this pattern is abolished in HET TAC mice (b). Note the irregular shapes and the distended and bullous appearance of single mitochondria in HET TAC (arrows). Asterisk: mitochondrium, mf: myofibrils, bar: 1.5μm.

To further assess the mitochondrial pathology found in HET TAC at the ultrastructural level, we performed transmission electron microscopy of left ventricular heart samples. In contrast to WT mice after TAC, we found that mitochondria in HET mice after TAC appeared distended and bullous in conjunction with a loss of their string-of-pearls-like distribution alongside the myofibrils (Fig 8).

## Discussion

Desminopathies result in numerous deleterious cardiac disease manifestations including various forms of cardiomyopathies, cardiac conduction defects, arrhythmias, and sudden cardiac death. Since no specific curative treatment options exist so far, there is an urgent need to identify factors that may aggravate or ameliorate the disease progression. We here investigated the impact of pressure overload to the heart in a *DES*-p.R349P knock-in mouse strain serving as a model for human autosomal-dominant desminopathies. Our study offers two major findings: First, pressure overload to a heart affected by the heterozygous *DES*-p.R350P mutation results in an accelerated development of cardiomyopathy and marked deterioration of cardiac function. Second, on cardiac tissue level the induction of pressure overload led to a reduced content of mtDNA copy numbers and to reductions of mitochondrial enzyme activities in the presence of a p.R350P mutant desmin protein.

Our experiments were performed in mice with and without induced pressure overload to investigate the pathophysiological effect of the *DES*-p.R350P mutation. All experiments were performed in adult mice that at this age and the absence of TAC-induced pressure overload did not exhibit any functional signs of cardiomyopathy. This is consistent with our previous work showing that pathological functional parameters like reduced left ventricular ejection fraction in desminopathy mice could not be detected before the age of two years [1]. Notably, our present study revealed that pressure overload in heterozygous desminopathy mice led to a more pronounced deterioration of cardiac function as compared to WT mice after TAC operation. In a previous study employing healthy mice, pressure overload resulted in only mild changes after two weeks, whereas after approximately five weeks the left ventricular ejection fraction was significantly reduced [26]. In contrast, here we could demonstrate a reduction of left ventricular ejection fraction and cardiac output as well as a significant increase in intercellular myocardial fibrosis two weeks after TAC in heterozygous *DES*-p.R349P knock-in mice as compared to WT littermates.

Telemetric ECG monitoring revealed that HET TAC animals, unlike WT TAC, did not show an increase in heart rate compared with the corresponding sham animals. This points towards an insufficient cardiac adaptive mechanism after pressure overload in our HET TAC animals. Consistent with the finding in our previous work, already sham treated HET mice showed an elevated susceptibility to cardiac arrhythmias, spontaneous VPCs, and AV blocks [1]. After the induction of pressure overload, however, HET mice now responded with an additional increase in the inducibility of atrial fibrillation and ventricular tachycardias, and marked increases in AV block durations. This can be explained well by the presence of structural changes including DCM [27] and fibrosis [28] which were also present in our desminopathy mice.

Our *DES*-p.R349P mice showed an elevated rate of inducible arrhythmia. Although most *DES* mutations with cardiac involvement reported to date result in a dilated cardiomyopathy phenotype, the development of an arrhythmogenic right ventricular cardiomyopathy as also been reported [6]. In these cases, a direct effect of desmin on the structure of the intercalated disks or desmosomes was assumed by analogy with true ARVC. However, also patients with dilated cardiomyopathy suffer from malignant ventricular arrhythmia. Sudden cardiac death, presumably due to ventricular fibrillation, is a common cause of death in this patient population. One main reason seems to be the cardiac dilatation with intercellular fibrosis and hence slowing of electrical impulse propagation [1]. The latter could convincingly be demonstrated in the present study.

Defects in the aggregation of ion channels as a result of structural perturbance of sarcolemma, cytoskeleton, and sarcomere represent a common pathomechanism for arrhythmogenesis in many cases of DCM [29]. Furthermore, the presence of mitochondrial dysfunction has recently been shown to promote arrhythmias [30]. In this setting, arrhythmias associated with mitochondrial dysfunction have been proposed to result from disturbances in the cardiac calcium metabolism. Additionally, impaired calcium homeostasis has been shown to occur in DCM [31, 32] and this might also contribute to the arrhythmic phenotype in our HET animals.

In diseased skeletal muscle, we previously demonstrated that murine *DES*-p.R349P and human *DES*-p.R350P both lead to a disruption of the extrasarcomeric desmin cytoskeleton and substantially perturb mitochondrial morphology, function and maintenance [16]. After TAC operation, solely the HET animals displayed reduced mitochondrial content, mtDNA copy numbers, and enzymatic activities. The latter findings imply a reduced number of functional mitochondria in the hearts of HET TAC desminopathy mice due to an insufficient adaption to the increased ATP demand in the mechanically stressed tissue potentially by increased mitophagy. The decreased mitochondria content is also in line with the severe myocardial fibrosis observed in these mice. In this context, it is noteworthy that the development of DCM with eccentric remodeling after left ventricular overload has been shown to result in enhanced mitochondrial apoptosis and impaired mitochondrial function which, however, only took place after a post-intervention period of eight weeks in healthy rats [33]. Moreover, decreases in respiratory chain function after pressure overload to the heart only take place with the beginning of impaired left ventricular function [34]. As a translational aspect in human desminopathies, our findings imply that the early initiation of antihypertensive therapies may be beneficial to prevent deterioration of cardiac function.

### Limitations

Implementation of TAC does not represent a physiological equivalence of arterial hypertension. However, with regard to the investigational setting, TAC facilitates more comparable results of pressure overload to the heart as a hypertensive drug treatment using osmotic mini-pumps for several weeks for example. To tackle a putative gender bias, we equally divided male and female animals in each group. As a result of a limited number of animals in the subgroups, we are not able to evaluate gender specific effects of the mutation. Also, we cannot answer the question if the observed changes in mitochondrial function are directly or indirectly related to the presence of the *DES-*p.R349P mutation *per se*. Further studies are also needed to clarify if other disease-causing desmin mutations exert a similar effect. As an alternative explanation, the mitochondrial changes could be a consequence of the structural and functional progression of heart failure.

## Conclusions

When subjected to pressure overload, heterozygous *DES*-p.R349P knock-in mice develop marked signs of cardiomyopathy with accompanying arrhythmias and AV blocks. These disease manifestations are associated with reductions in mtDNA copy numbers and mitochondrial enzyme activities implying a reduced number of mitochondria. In particular, early initiation of antihypertensive therapy in human p.R350P desminopathy patients is reasonable and might represent a crucial therapeutic measure to prevent cardiac deterioration. Furthermore, repeated monitoring of arterial blood pressure and consequent antihypertensive therapy seems to be advisable in desminopathy patients in general.

## Supporting information

S1 Fig Heart weight/tibia length. a) TAC operation did not significantly alter the heart weights in both genotypes. b) Tibia length did not differ between the investigated groups. b) The heart weight / tibia length ratio was highest in HET-TAC mice indicating cardiac hypertrophy. * = p<0.05 with Tukey’s Multiple Comparison Test.
(TIFF)

S2 Fig Uncropped original blot for “mtDNA long-range PCR (Fig 7). (TIF)

S1 File Original raw data. (XLSX)
