# Supplementary figures and images for: Heart failure after pressure overload in autosomal-dominant desminopathies: Lessons from heterozygous DES-p.R349P knock-in mice
Source: PLoS One. 2020 Mar 3;15(3):e0228913. doi: 10.1371/journal.pone.0228913 (PMC7053759; doi:10.1371/journal.pone.0228913)

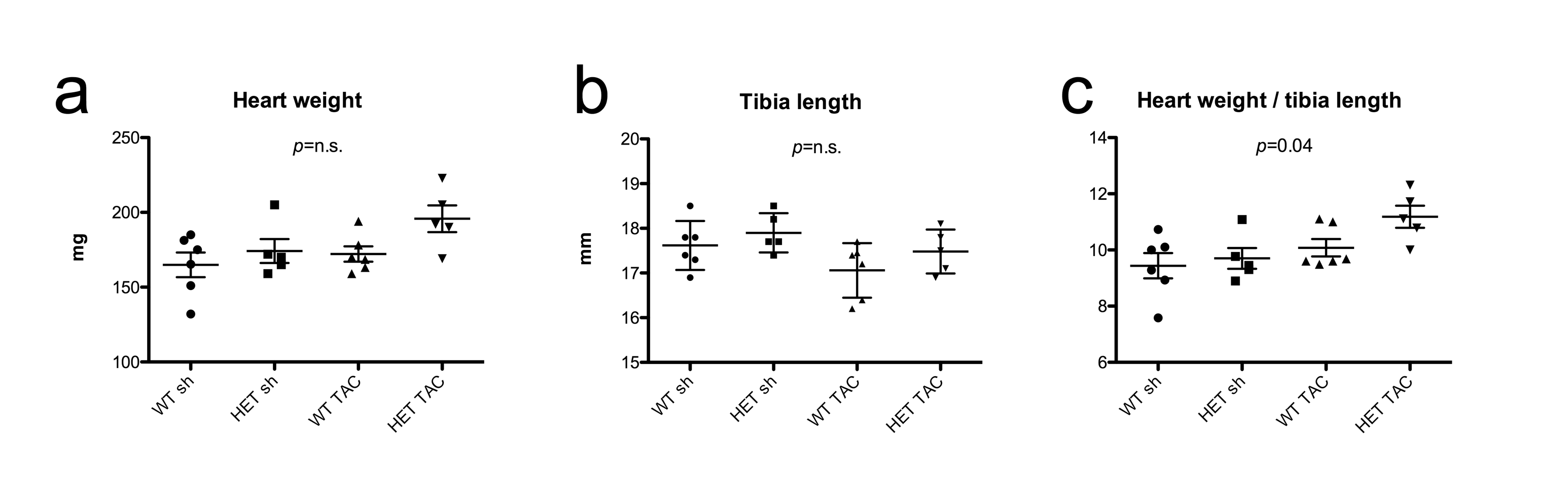

Supplement: S1 Fig — a) TAC operation did not significantly alter the heart weights in both genotypes. b) Tibia length did not differ between the investigated groups. b) The heart weight / tibia length ratio was highest in HET-TAC mice indicating cardiac hypertrophy. * = p<0.05 with Tukey’s Multiple Comparison Test. (TIFF) [file pone.0228913.s001.tiff]

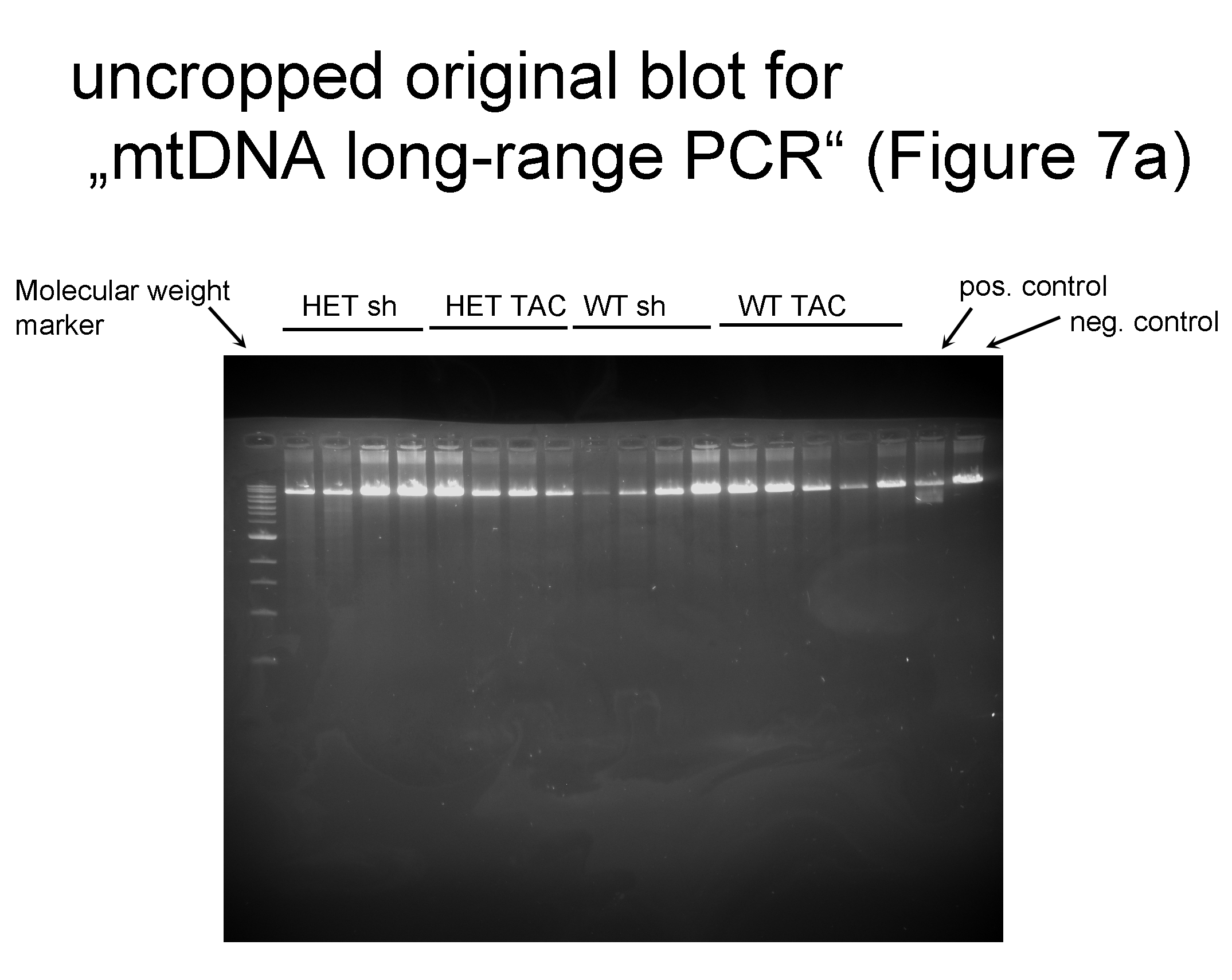

Supplement: S2 Fig — (TIF) [file pone.0228913.s002.tif]
